# Draft Genome Sequence of *Prosthecomicrobium hirschii* ATCC 27832^T^

**DOI:** 10.1128/genomeA.01355-15

**Published:** 2015-11-19

**Authors:** Jeremy J. Daniel, Scott A. Givan, Yves V. Brun, Pamela J. B. Brown

**Affiliations:** a Division of Biological Sciences, University of Missouri, Columbia, Missouri, USA; b Informatics Research Core Facility, University of Missouri, Columbia, Missouri, USA; c Department of Molecular Microbiology and Immunology, University of Missouri, Columbia, Missouri, USA; d Department of Biology, Indiana University, Bloomington, Indiana, USA

## Abstract

We report the draft genome sequence of *Prosthecomicrobium hirschii* ATCC 27832^T^, an alphaproteobacterium with remarkable cellular morphologies. The chromosome comprises 6,484,983 bp in six scaffolds with a G+C content of 69%, and 6,066 potential coding sequences.

## GENOME ANNOUNCEMENT

The alphaproteobacterium *Prosthecomicrobium hirschii* produces two morphologically distinct cell types; short-stalked cells produce numerous short conical stalks, whereas long-stalked cells typically have fewer than eight long cylindrical stalks (1). The life cycle of this bacterium is complex (1). Typically, each cell type continues to produce that same morphotype for many generations. However, shifts in cell type can occur via polar growth and subsequent cell division, presumably in response to changing environmental conditions. *P. hirschii* cells grow by budding in which new peptidoglycan synthesis occurs at the cell pole (1, 2), presumably enabling a mother cell to produce a daughter cell of a different morphotype. The genome sequencing of *P. hirschii* was initiated to facilitate an enhanced understanding of the complex dimorphic life cycle and the mechanism of cell growth in this bacterium.

The draft *P. hirschii* genome was sequenced using standard paired-end 454 protocols at the Center for Genomics and Bioinformatics at Indiana University and using standard Illumina sequencing protocols on MiSeq at the University of Missouri DNA Core Facility. A hybrid assembly using MaSuRCA version 2.3.2b (3) generated six contigs comprising 6,484,983 bp. The draft genome sequence was annotated using the NCBI prokaryotic genome annotation pipeline (4, 5). The genome is predicted to contain a total of 6,066 coding sequences (CDSs) encompassing 5,692,356 nucleotides giving an 87.8% coding percentage; 4,345 CDSs (71.6%) contain at least one sequence in the COGs database.

Preliminary analysis of the genome sequence of *P. hirschii* indicates that this organism possesses a set of genes involved in the regulation of cell cycle progression in alphaproteobacteria (6), suggesting that this organism may exhibit a *Caulobacter*-like cell cycle. In addition, a putative operon predicted to encode a LuxR-type transcriptional regulator and a LuxI-like autoinducer synthase (7) are also present, indicating that *P. hirschii* may use quorum sensing to regulate some physiological activities. Further analysis of this genome should provide insights into the evolution of diverse bacterial cell shapes, bacterial cell cycles, and mechanisms of cell growth.

### Nucleotide sequence accession number.

The draft genome sequence of *P. hirschii* ATCC 27832^T^ has been deposited in DDBJ/ENA/GenBank under the accession number LJYW00000000.
